# Long term results after surgical management of posterior wall acetabular fractures

**DOI:** 10.1007/s10195-014-0297-8

**Published:** 2014-05-31

**Authors:** Narender Kumar Magu, Paritosh Gogna, Amanpreet Singh, Rohit Singla, Rajesh Rohilla, Amit Batra, Reetadyuti Mukhopadhyay

**Affiliations:** Department of Orthopaedics and Rehabilitation, PGIMS, 2/11-J (UH) Medical Enclave, Rohtak, Haryana 24001 India

**Keywords:** Acetabular fracture, Posterior wall fracture of the acetabulum, Long term outcome

## Abstract

**Background:**

Posterior wall fractures are the most common of all acetabular fractures, and there is universal consensus that displaced fractures are best treated with anatomical reduction and stable internal fixation. Though early and mid term results for such studies are available, few shed light on long term results. This study was performed to evaluate long term functional and radiological outcomes in patients with posterior wall acetabular fractures and to determine factors that may contribute adversely to a satisfactory final outcome.

**Materials and methods:**

We retrospectively analysed the hospital records for patients who underwent open reduction and internal fixation (ORIF) for posterior wall acetabular fractures. Twenty-five patients (20 men, five women), including one with bilateral posterior wall fracture, with a mean age of 41.28 ± 7.16 years (range 25–60 years) and a mean follow-up of 12.92 ± 6.36 years (range 5–22 years) who met the inclusion criteria formed the study cohort. Matta’s criteria were used to grade postoperative reduction and final radiological outcome. Functional outcome at final follow-up was assessed according to d’Aubigné and Postel score.

**Results:**

Anatomic reduction was achieved in 22 hips, imperfect in four and poor in none. Radiological outcome at final follow-up revealed excellent results in ten hips, good in eight, fair in five and poor in three. The final d’Aubigné and Postel scores were excellent in 14 hips, good in six and fair and poor in three each. Patients with anatomical reduction had a favourable functional and radiological long term outcome. However, the presence of associated injuries in lower limbs and a body mass index (BMI) >25 adversely affected the final functional outcome. Osteonecrosis was seen in three patients, heterotopic ossification in two and Morel Lavallee lesion in one. One patient had postoperative sciatic nerve palsy, which recovered 6 weeks after surgery.

**Conclusion:**

Anatomic postoperative reduction leads to optimal functional and radiological outcome on long term follow-up; however, the presence of associated lower-limb injuries and BMI >25 adversely affects a satisfactory final outcome in patients with posterior wall acetabular fractures.

**Level of evidence:**

(Level 4) Retrospective case series.

## Introduction

Fracture of the acetabular posterior wall accounts for approximately one fourth to one third of all acetabular fractures [1–3]. Displaced acetabular fractures are best treated with anatomical reduction and stable internal fixation. The goal of operative treatment is to achieve precise anatomical reduction to attain a painless, mobile and stable hip. The long term results of operative treatment are influenced by numerous factors, including fracture type and/or dislocation, femoral-head status, intra-articular osteochondral fragments, injury duration, reduction quality, local complications, associated injuries and surgical approach [4]. Osteoarthrosis of the hip joint, avascular necrosis (AVN) of the femoral head and heterotopic ossification tend to result in poorer outcome despite good fracture reduction [5, 6]. The purpose of this was to evaluate long term functional and radiologic outcomes in patients with posterior wall acetabular fractures to determine factors that may contribute adversely to satisfactory final outcome and to identify clinical situations that may be overlooked initially but may have serious consequences on final outcome.

## Materials and methods

We retrospectively reviewed hospital records of patients who underwent open reduction and internal fixation (ORIF) for posterior wall acetabular fractures between 1990 and 2007. The radiographs and computed tomography (CT) scans were studied, and the fracture was classified as per Judet et al. [7]. Patients sustaining fractures other than in the posterior wall, who presented >2 weeks after injury, had stable/undisplaced fractures and those without Judet’s radiographs were excluded. Twenty-five patients, including one with bilateral hip involvement, fulfilled inclusion criteria and formed the patient cohort. The treatment protocol for fractures and hip dislocation initially involved closed reduction under sedation/anaesthesia, followed by upper tibial skeletal traction, with weights raging from 7.5 to 10 kg. Eighteen patients had associated posterior dislocation, which was reduced within 12 h of injury in 12 patients, between 12 and 24 h in five and after 24 h in one.

All patients had plain pelvic radiographs (anteroposterior, and two 45° oblique Judet views). All patients were operated using the Kocher–Langenbeck surgical approach in a floppy lateral position [8]. In all cases, the sciatic nerve was first identified and protected after tracing it proximally and medially towards the greater sciatic notch. The operating surgeon used his fingertips to retract the sciatic nerve during the surgical procedure and no nerve retractor was used. Ganz trochanteric flip osteotomy was done in three cases to allow for “sliding forward” of gluteus medius/minimus to expose the superior aspect of the acetabulum when the fracture involved the roof or if a fragment was displaced towards the anterior inferior iliac spine under the gluteus medius muscle [9]. Care was taken to preserve soft-tissue attachments to the displaced posterior fragment. Posterior wall fractures were reduced anatomically and appropriately stabilised temporarily with Kirschner wires, followed by definitive fixation either with 4-mm cancellous lag screws (*n* = 9), 6.5-mm cancellous lag screw (*n* = 1) or interfragmentary screws and a reconstruction plate (*n* = 16). Stabilisation with screws alone was strictly reserved for fractures consisting of one large posterior wall fragment and was buttressed with a 3.5-mm reconstruction plate whenever the fracture was comminuted. Loose intra-articular bony fragments were removed using a distractor under direct vision without further redislocating the joint. Operative findings included free intra-articular osteochondral fragments in three patients, injury to the femoral-head articular surface in two, acetabular articular impaction (marginal impaction) in two, massive posterior wall fracture comminution in eight, anterior migration of postwall fragment (during close reduction) in two and post-wall rotation to 180° in two. When marginal impaction was present, the impacted articular cartilage was elevated and reduced to its anatomic position over the femoral head, which served as a template. The defect so created was filled with cancellous grafts taken from the greater trochanter. Closed-suction surgical drains were used for 24–72 h. Though prophylactic antibiotics were used during the perioperative period, no prophylaxis against heterotopic ossification (indomethacin or radiation) or deep venous thrombosis was used. Patients were taught and encouraged to perform intermittent, pain-free quadriceps-, hip- and knee-flexion exercises with traction starting on the second postoperative day. Partial weight bearing was permitted 6 weeks after surgery, gradually progressing to full weight bearing at 12 weeks.

Fracture reduction was evaluated by measuring residual displacements on the three postoperative radiographs (anteroposterior and two 45° oblique Judet views) according to criteria developed by Matta [4]. According to this criteria postoperative reduction was graded as anatomical (0–1 mm of displacement), imperfect (2- to 3-mm of displacement) or poor (>3-mm displacement). The final follow-up radiographs were graded according to Matta [4]. An excellent grade was given to a normal-appearing hip joint, good to mild for minimal sclerosis and joint narrowing, fair to intermediate for moderate sclerosis and joint narrowing (<50 %) and poor for greater changes. At the final follow-up, functional outcome was evaluated using a modification of the clinical grading system developed by d’Aubigné and Postel [10]. AVN of the femoral head was classified according to Ficat and Arlet [11]. Heterotopic ossification was graded according to Brooker et al. [12]. Fisher’s exact test was used to compare postoperative reduction quality with functional and radiological outcome at the time of final follow-up and to identify the effect of Quetelet index (BMI) and presence of associated injuries in the lower limb on final functional outcome.

## Results

**Fig. 1:**
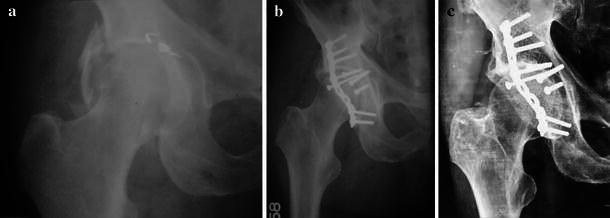
**a** Preoperative anteroposterior (AP) radiographs of a 40-year-old man showing posterior acetabular-wall fracture, **b** postoperative AP radiograph showing anatomical reduction, **c** AP radiographs at 22 years’ follow-up showing excellent radiological outcome

**Fig. 2:**
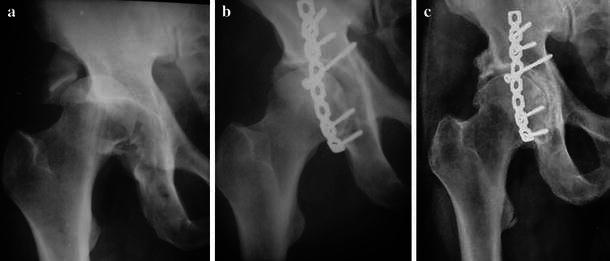
**a** Preoperative anteroposterior (AP) radiographs of a 42-year-old man showing posterior acetabular-wall fracture and associated hip-joint dislocation, **b** postoperative AP radiograph showing anatomical reduction, **c** AP radiographs at 20 years’ follow-up showing minimal sclerosis with mild osteoarthritic changes

**Fig. 3:**
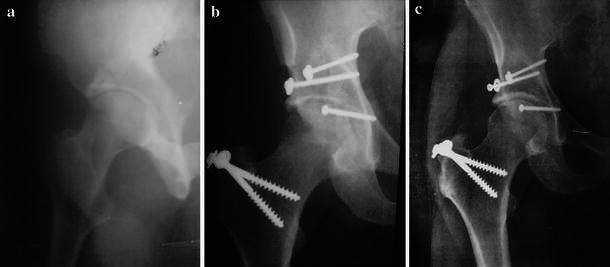
**a** Preoperative anteroposterior (AP) radiographs of a 27-year-old man showing posterior acetabular-wall fracture, **b** osteosynthesis of the posterior wall using lag screws and trochanteric flip osteotomy; postoperative AP radiographs showing anatomical reduction, **c** AP radiograph at 10-years’ follow-up showing excellent radiological outcome

Mean patient age (20 men, five women) was 41 ± 7.16 years (range 25–60 years). The right acetabulum was involved in 17 patients and the left in seven; one had bilateral hip involvement. The mode of injury was road-traffic accident in 20 and fall from a height in five. The associated injuries were present in ten patients, which included lower-extremity injuries in six patients (contralateral acetabulum *n* = 1, ipsilateral femoral shaft *n* = 2, contralateral femoral shaft *n* = 1, tibial plateau *n* = 1, contralateral femoral-head fracture *n* = 1), upper-extremity trauma (distal radius *n* = 3, proximal humerus *n* = 1). Average follow-up was 12.92 ± 6.36 years (range 5–22 years), average time between injury and surgical procedure 4.2 ± 1.7 days (range 3–12 days) and average operative time 105 min (range 100–120 min). Fracture-reduction quality postoperatively, as measured on plain radiographs, was graded as anatomic in 22 hips, imperfect in four and poor in none (Table 1). At final follow-up, radiographic outcome according to Matta [4] revealed excellent results in ten hips, good in eight, fair in five and poor in three. Final d’Aubigné and Postel scores were excellent in 14 hips, good in six, fair in three and poor in three (Figs. 1, 2, 3).

**Table 1:** Clinicoradiological workup of patients with acetabular fractures

Sr.	Sex/age (years)	Postoperative reduction	Associated lower-limb injury	BM1	Complications	Follow-up (years)	Final radiological outcome	Final d’Aubigné and Postel scores
1	Male, 37	Anatomical	Tibial plateau^a^ I/L	25	–	22	Good	Good
2	Male, 40	Anatomical	–	23	–	22	Excellent	Excellent
3	Male, 42	Anatomical	–	21	–	22	Excellent	Excellent
4	Male, 38	Anatomical	–	22	–	21	Good	Excellent
5	Male,42	Imperfect	–	32	AVN (THR)	21	Poor	Poor
6	Male, 42	Anatomical	–	24		20	Good	Good
7	Male, 52	Imperfect	–	21	–	20	Fair	Fair
8	Male, 48	Imperfect	–	23	AVN	20	Poor	Poor
9	Male, 32	Anatomical	–	23	Grade II HO	14	Good	Excellent
10	Male, 33	Anatomical	–	23	–	13	Excellent	Excellent
11	Male, 42	Anatomical	–	22	–	13	Excellent	Excellent
12	Male, 45	Anatomical	C/L^a^ SOF	23	–	13	Good	Excellent
13	Male, 46	Anatomical	–	24	Morel–Lavallee lesion	13	Excellent	Excellent
14	Male, 42	Anatomical	Both acetabulum	28	–	12	Fair	Fair
15	Female, 25	Anatomical	I/L^a^ SOF	22	–	12	Fair	Good
16	Male, 27	Anatomical	–	23	–	10	Excellent	Excellent
17	Male, 42	Anatomical	C/L femoral head	24	–	8	Good	Good
18	Female, 45	Anatomical	–	22	–	8	Excellent	Excellent
19	Male, 42	Anatomical	–	22	–	7	Excellent	Excellent
20	Female, 43	Anatomical	–	20	–	7	Excellent	Excellent
21	Male, 60	Anatomical	–	22	Postoperative sciatic neuropraxia	5	Good	Excellent
22	Male, 44	Imperfect	–	22	Grade II HO	5	Fair	Good
23	Female, 41	Anatomical	–	24	–	5	Excellent	Excellent
24	Male, 42	Anatomical	I/L^a^ SOF	22	AVN	5	Poor	Poor
25	Female, 40	Anatomical	–	21	–	5	Good	Good

*BMI* body mass index, *SOF* shaft of femur, *C/L* contralateral, *I/L* ipsilateral, *HO* heterotopic ossification, *AVN* avascular necrosis, *THR* total hip arthroplasty

^a^ Fracture

Fisher’s exact test revealed that anatomical fracture reduction resulted in better long term radiological outcome compared with nonanatomical reduction (*p* = 0.0047). It also showed that anatomical reduction was associated with better long term functional outcome compared with nonanatomical reduction (*p* = 0.0278). In patients with anatomical reduction, the presence of associated injuries in lower limbs adversely affected final functional outcome compared with isolated posterior wall fracture (*p* = 0.0198), as did BMI >25 compared with BMI <25 (*p* = 0.0308). At the final review, osteonecrosis was seen in three patients: two grade III and grade IV changes. The latter patient underwent total hip replacement and was asymptomatic at the final follow-up. One patient had Morel–Lavallee lesion and was managed with multiple stab incisions and negative suction drain, along with antibiotics and daily redressing. No patient had deep infection, recurrent dislocation, pulmonary embolism or revision fixation. Grade II heterotopic ossification was seen in two patients (Table 2). One patient had postoperative sciatic nerve palsy, which recovered 6 weeks after surgery. Preoperative neurologic deficit was present in one patient, which recovered postoperatively.

**Table 2:** Radiological and functional outcome of patients at final follow-up

Fracture reduction	Radiological outcome				Functional outcome				AVN	HO
	Excellent	Good	Fair	Poor	Excellent	Good	Fair	Poor		
Anatomical	10	8	3	1	14	5	2	1	1	1
Imperfect	–	–	2	2	–	1	1	2	2	1

*AVN* avascular necrosis, *HO* heterotopic ossification

## Discussion

The main findings of this study were that patients with anatomical reduction have a favourable functional and radiological outcome on a long term basis. However, in patients with anatomical reduction, the presence of associated injuries in lower limbs and a BMI >25 adversely affected the final functional outcome. An anatomical reduction was achieved in 84.61 % patients, which is comparable with the rates 80–90 % reported in the literature [4, 13]. We resorted to screw fixation alone wherever fracture configuration comprised a large, solid, single chunk of bone (*n* = 10); plate-and-screw fixation was used in the remainder of cases (*n* = 16). Screw fixation permits a lesser degree of soft-tissue handling and dissection compared with plate-and-screw fixation. Im et al. [14] obtained excellent to good results in 14 of 15 patients using fixation with lag screws and proposed that the screw facilitates reduction and minimises soft-tissue dissection; in our study, we used minimal soft-tissue stripping, which led to favourable outcome [15]. Soft-tissue-sparing using the modified Kocher–Langenbeck approach involves working on the posterior wall through windows between the gluteus medius and piriformis muscles superiorly and between short rotators and ischial tuberosity inferiorly without dividing the rotators and abductors [15].

Articular congruity reconstruction and stable fixation reduces the incidence of posttraumatic osteoarthritis. The rate of symptomatic posttraumatic arthritis was 23.07 % in our patients and is reported to be 9–24 % in other series [16, 17]. We emphasise, however, the relevance of associated injuries in lower limbs and a high BMI, which are important contributing factors in long term functional outcome. Two of our patients with anatomical reduction on postoperative X-rays and full range of motion (ROM) began experiencing persistent hip pain that interfered with activities of daily living at 6 and 9 years after surgery: one had a BMI >25 and the other associated tibial plateau fracture malunion; radiographic follow-up revealed the development of osteoarthritic changes, which gradually progressed till final follow-up. Such patients have an increased propensity to early development of osteoarthritic changes that are not contributable to the primary fracture but to associated injuries of the lower limb and high BMI. Thus, a high percentage of long term good-to-excellent results can be expected following anatomic reduction and stable internal fixation of these fractures, although anatomical reduction is not the sole criteria for a good final outcome.

Three patients had femoral-head AVN, the reported incidence after acetabular fractures being 10–15 % [18, 19], which is in consensus with an incidence of 11.53 % in our study. Six patients underwent delayed reduction of an associated dislocated hip (>12 h); two developed AVN. The third case of AVN was in a patient who had iatrogenic medial circumflex femoral artery (MCFA) injury during surgery. Although AVN development has been reported as late as 8 years after surgery [20], all three cases in our series presented within 3 years of injury. We therefore suggest that all patients be followed closely for at least 3 years for AVN development.

Heterotopic ossification occurs most frequently in patients in whom gluteal muscles are dissected, and necrotic gluteus minimus muscle resection diminishes heterotopic ossification formation [21]. Though grade II heterotopic ossification was seen in 7.69 % of our hips, no case was severe despite not using prophylaxis. Some patients with a 22-year follow-up showed a lower incidence of heterotopic ossification compared with that reported in the literature [22–25]; in our patients, stride was short, terminal movements only were restricted and overall hip-joint function was not greatly affected. In another study, only one case of Brooker class II heterotopic ossification was observed in a series of 14 patients in whom a modified approach was used; that patient required trochanteric flip osteotomy [15]. Thus, attempting to preserve soft tissue is important. We found one case of sciatic-nerve neurapraxia, which may have been due to excessive traction during surgery; this resolved 6 weeks postoperatively. We urge careful sciatic nerve retraction using fingers of free hand, not retractors.

Two important clinical situations in acetabular posterior wall surgery may be overlooked but may have serious consequences on final outcome. A 180° rotation of the noncongruous fragment of the acetabular dome is often associated with posterior acetabular-wall fractures. It is important to address this situation lest the patient may develop restricted ROM, thus interfering with activities of daily living and causing osteoarthritic changes in the long term. Also, inferior posterior wall fragments without soft-tissue attachment may be discarded, as there is a possibility they will not incorporate during healing and, instead, act as irritative loose bodies, which may enter the joint and perpetuate osteoarthritic changes. Removing such fragments does not lead to instability. We encountered such a situation in two patients, and at final follow-up of 8 and 10 years, the hip remained stable.

Our study is somewhat limited by its retrospective nature and relatively small population size. However, these are limitations in most series on posterior acetabular-wall fractures [26]. Its strength is that it is a single-institution study, with all cases operated by the same surgical team and with an unusually long follow-up. To conclude, anatomic reduction leads to optimal long term functional and radiologic outcomes in patients with fractures of posterior acetabular wall. However, even in patients with anatomical reduction, the presence of associated injuries in the lower limb and a BMI >25 adversely affect final functional outcome. Associated injuries in lower limbs should be tackled meticulously, and patients with a high BMI should be informed about the possible deterioration of function over the long term and be encouraged to lose weight. Long term results in our study are quite encouraging and are in favour of anatomical reduction of these fractures.
